# Potential Locations for Noninvasive Brain Stimulation in Treating Autism Spectrum Disorders—A Functional Connectivity Study

**DOI:** 10.3389/fpsyt.2020.00388

**Published:** 2020-05-07

**Authors:** Yiting Huang, Binlong Zhang, Jin Cao, Siyi Yu, Georgia Wilson, Joel Park, Jian Kong

**Affiliations:** Department of Psychiatry, Massachusetts General Hospital, Harvard Medical School, Boston, MA, United States

**Keywords:** autism spectrum disorders, meta-analysis, fMRI, noninvasive brain stimulation

## Abstract

**Objectives:**

Noninvasive brain stimulation (NIBS) is an emerging tool for treating autism spectrum disorder (ASD). Exploring new stimulation targets may improve the efficacy of NIBS for ASD.

**Materials and Methods:**

We first conducted a meta-analysis on 170 functional magnetic resonance imaging studies to identify ASD-associated brain regions. We then performed resting state functional connectivity analysis on 70 individuals with ASD to investigate brain surface regions correlated with these ASD-associated regions and identify potential NIBS targets for ASD.

**Results:**

We found that the medial prefrontal cortex, angular gyrus, dorsal lateral prefrontal cortex, inferior frontal gyrus, superior parietal lobe, postcentral gyrus, precentral gyrus, middle temporal gyrus, superior temporal sulcus, lateral occipital cortex, and supplementary motor area/paracentral gyrus are potential locations for NIBS in ASD.

**Conclusion:**

Our findings may shed light on the development of new NIBS targets for ASD.

## Introduction

Autism spectrum disorder (ASD) is a highly prevalent disorder (1). Despite decades of research, the treatment of ASD is far from satisfactory (2). To date, only a handful of treatment options have been shown to ameliorate the symptoms associated with the disorder (3). Developing new intervention methods for ASD is therefore urgently needed.

Noninvasive brain stimulation (NIBS) is an emerging tool for the treatment of ASD. Such a technique may include repetitive transcranial magnetic stimulation (rTMS), transcranial direct current stimulation (tDCS), and transcranial alternating current stimulation (tACS) (4, 5). Preliminary evidence has demonstrated the potentials of rTMS and tDCS in the treatment of ASD (6–8). However, results from recent meta-analyses regarding the effectiveness of these NIBS techniques in ASD treatment are inconsistent (9–11), necessitating that researchers further investigate and improve NIBS treatment.

Refining stimulation targets may be a promising way to improve the treatment effect of NIBS. Currently, multiple NIBS targets for ASD treatment have been explored, including the dorsal lateral prefrontal cortex (DLPFC), motor cortex, inferior frontal gyrus (IFG), dorsal medial prefrontal cortex (dmPFC), and temporoparietal junction (TPJ) (9, 10, 12–14). However, the rationale for choosing these areas is often ambiguous, significantly limiting the optimization of NIBS and its application in ASD.

Neuroimaging studies have discovered a large number of ASD-associated brain regions and have expanded our understanding of ASD pathophysiology (15–18). However, clinical translation is still limited. One way of selecting target brain regions for NIBS from neuroimaging research is through meta-analysis of previous studies. However, some brain regions identified from these meta-analyses, such as the fusiform gyrus and amygdala, are located in areas that are inaccessible for certain NIBS technologies such as tDCS.

Recently, investigators have started to apply resting state functional connectivity methods to optimize the locations of NIBS for depression treatment and have achieved some encouraging results, demonstrating the potential of resting state functional connectivity in refining NIBS target locations (19–21). Nevertheless, few studies have systematically investigated potential locations for ASD using brain imaging. This study aims to explore new potential target locations for NIBS in ASD treatment by combining meta-analysis and resting state functional connectivity methods. Specifically, we first performed an automated meta-analysis of ASD and defined regions of interest (ROI). Then, we investigated the resting state functional connectivity of those ROIs in 70 ASD patients to identify easily accessible locations for NIBS, particularly tDCS.

## Materials and Methods

### Identifying ASD-Associated ROIs From the Meta-Analysis

To extract ASD-associated ROIs, we used Neurosynth (22) (http://neurosynth.org/; accessed 11 March 2020) as a metadata reference of the neuroimaging literature. Under the search string “autism spectrum,” 170 fMRI studies were identified, and a uniformity test map was generated to identify ASD-associated brain regions. A complete list of the 170 fMRI studies extracted from Neurosynth can be found in **Supplementary Material Table S1**.

To create ASD-associated ROIs for further analysis, we first used xjView toolbox (http://www.alivelearn.net/xjview/) to identify the coordinates with peak z-scores within all clusters larger than 50 voxels on the uniformity test map. Then, 6-mm radius spherical masks centered on the identified peak coordinates were created using WFU_PickAtlas toolbox (version 3.0.5b, http://fmri.wfubmc.edu/software/PickAtlas). The ROIs were further refined by taking the overlap of the uniformity test map with the whole brain cortical masks (see **Supplementary Material Figure S1**) from the WFU_PickAtlas for the purpose of maintaining regional specificity.

### Subjects and MRI Data Acquisition

Data were extracted from Autism Brain Imaging Data Exchange II. Subjects were selected from three data sites: Georgetown University, New York University (sample 1 and sample 2), and Kennedy Krieger Institute from Autism Brain Imaging Data Exchange (ABIDEII) (23). We selected subjects based on the following criteria: (1) 5–12 years old; (2) full-scale IQ (FIQ) scores > 80; (3) diagnosis of ASD based on DSM-IV-TR and assessed with the Autism Diagnostic Observation Schedule (ADOS) (24) and/or the Autism Diagnostic Interview–Revised (ADI-R) (25); (4) no history of attention-deficit hyperactivity disorder, oppositional defiant disorder, or phobia. All procedures from these three sites were approved by their local Institutional Review Boards.

The resting-state fMRI and high-resolution T1-weighted brain structural images were acquired on 3T MRI scanners (see details of the acquisition parameters in http://fcon_1000.projects.nitrc.org/indi/abide/abide_II.html).

### Image Preprocessing

The images were preprocessed in CONN version 18a (https://sites.google.com/view/conn/) (26) and SPM 12 (http://www.fil.ion.ucl.ac.uk/spm/) using CONN's default preprocessing pipeline. The preprocessing steps included slice-timing correction, realignment, normalization (3×3×3 mm^3^ in MNI space), and smoothing (6×6×6 mm^3^). During preprocessing, the Artifact Detection Tool (https://www.nitrc.org/projects/artifact_detect/) was used to detect outliers (> 3 SD and/or >0.5mm). The outliers were used for subsequent scrubbing regression. The structural images were segmented and used to create gray matter, white matter (WM), and cerebral spinal fluid (CSF) masks of each subject. Then, linear regression using WM & CSF signals (CompCor; 5 components for WM and CSF), linear trend, subject motion (six rotation/translation motion parameters and six first-order temporal derivatives), and outliers (scrubbing) was conducted to remove confounding effects. Afterwards, the residual BOLD time series were band-pass filtered (0.008–0.09 Hz).

### Functional Connectivity Analysis

Similar to our previous study (27, 28), to explore potential brain surface regions related to ASD, 21 ROIs identified from the meta-analysis were used to conduct seed-to-voxel functional connectivity analyses on resting-state fMRI data from all selected ASD subjects. The residual BOLD time course was extracted from the 21 ROIs, and Pearson's correlation coefficients were computed between the ROIs and all other brain voxels for each subject to create subject-level seed maps. The resulting correlation coefficients were subsequently transformed into z-scores to increase normality. At the group level, all subject-level seed maps were included in a one-sample t-test to obtain a group-level correlation map.

After the one-sample t-test, a brain surface mask was created to exclude brain regions that are not on the brain surface (inaccessible to NIBS). The regions included in the brain surface mask were the bilateral pre and postcentral gyri; superior and middle frontal gyri; superior, inferior, and middle occipital gyri; superior and inferior parietal lobules; supramarginal gyrus; angular gyrus; superior temporal gyrus; superior temporal pole; middle temporal gyrus; middle temporal pole; inferior temporal gyrus; opercular inferior frontal gyrus; Rolandic operculum; triangular inferior frontal gyrus; superior medial frontal gyrus; calcarine sulcus; orbital middle, superior, and inferior frontal gyri; orbital medial frontal gyrus; supplementary motor area; paracentral lobule; precuneus; and cuneus (see **Supplementary Material Figure S1** for detailed mask image).

### Exploring Potential NIBS Locations for ASD

**Figure 1:**
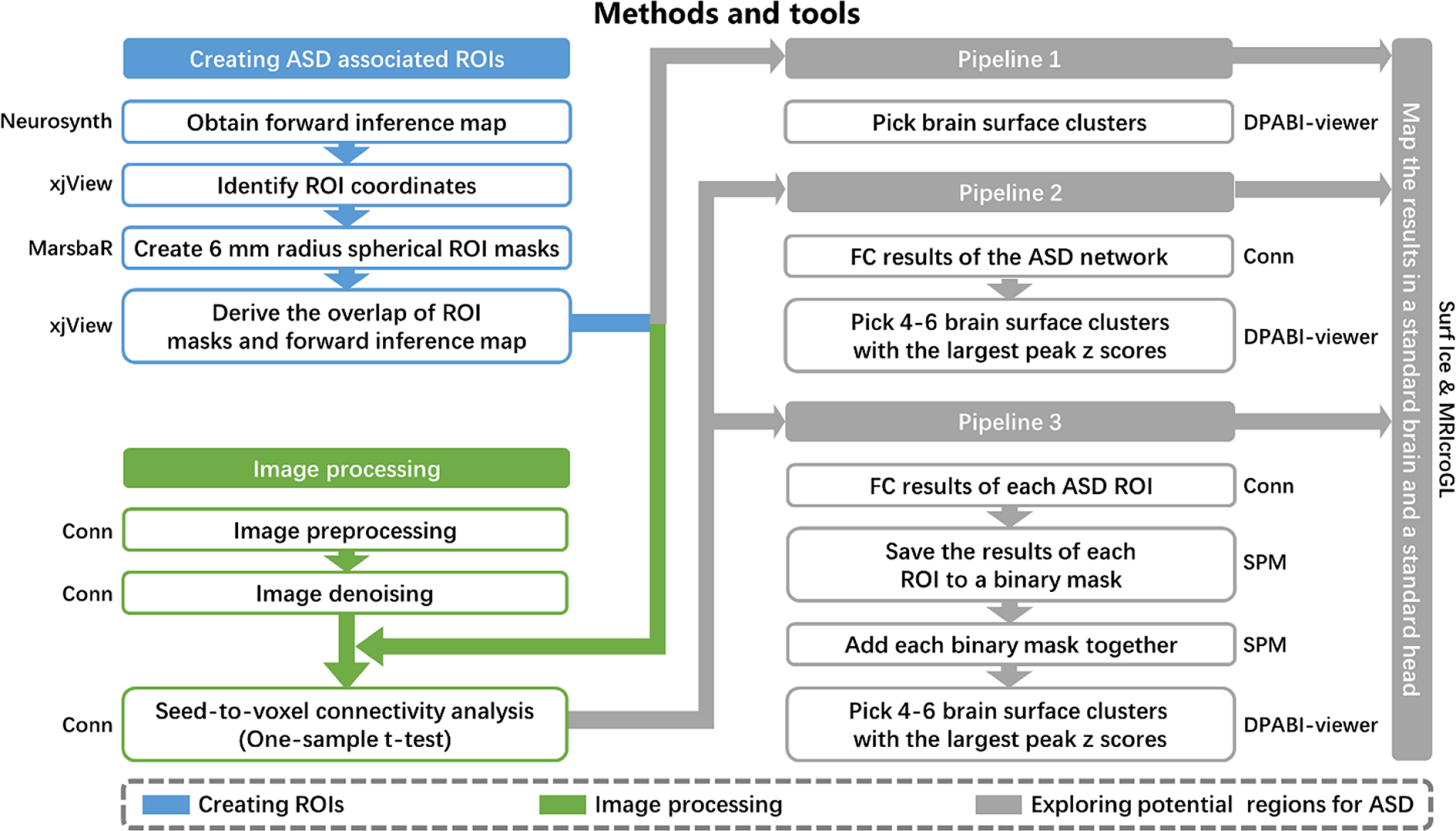
Methods and tools. ROI, regions of interest; ASD, autism spectrum disorder; FC, functional connectivity.

Similar to our previous study (27, 28), three different pipelines were applied to identify potential brain surface regions for NIBS in ASD (**Figure 1**). The most straightforward approach was pipeline 1, which used the meta-analysis to identify brain areas associated with ASD. Unfortunately, most of these brain areas are not located on the brain surface and therefore may be inaccessible to neuromodulation methods. We thus also employed pipelines 2 and 3 to identify surface brain areas that are functionally linked to deep brain structures associated with ASD, and hopefully stimulating these surface areas may influence the function of the deep brain areas.

Pipeline 1. We selected brain surface clusters in the uniformity test map from the meta-analysis by applying the whole brain cortical mask. These clusters represent potential brain surface regions (that are accessible to NIBS) directly involved in the pathophysiology of ASD.

Pipeline 2. The 21 refined ASD-associated ROIs from the meta-analysis were combined to form one ROI, which represented the ASD network for seed-to-voxel connectivity analysis in CONN. Then, we selected 4–6 brain surface clusters with the largest peak z-scores among all clusters with a voxel size larger than 50 and intensity larger than 4 on the group-level correlation map (positive and negative correlation maps separately). These clusters represent the brain surface regions that have the strongest correlations with the ASD functional network. A voxel-wise level threshold of p < 0.001 and a cluster level family-wise error (FWE) of p < 0.05 were applied to obtain group-level correlation maps of the ROIs.

Pipeline 3. We saved the group-level correlation maps of each ASD-associated ROI to a binary mask. The binary masks of all ROIs were added together to form a third-level map, positive correlation map, and negative correlation map separately. The intensity of each voxel in the third-level map may represent the number of ASD ROIs correlated to the voxel. Then, we selected 4–6 brain surface clusters with the largest peak z-scores among all clusters with a voxel size larger than 50 and intensity larger than 4 on the third-level correlation map as potential regions. These clusters represent the brain surface regions correlated with the largest number of ASD ROIs. A voxel-wise level threshold of p < 0.001 and a cluster level family-wise error (FWE) of p < 0.05 were applied to obtain group-level correlation maps of the ROIs.

The results from the three pipelines were mapped onto a standard brain using Surf Ice (https://www.nitrc.org/projects/surfice/) and a standard head using MRIcroGL (http://www.mccauslandcenter.sc.edu/mricrogl/) with the international 10-20 system in MNI space. The MNI coordinates of the 10-20 system were extracted from a previous study (29).

## Results

### Participant Demographics and Characteristics

Demographic and clinical characteristics of the study groups are summarized in **Table 1**. In total, 70 ASD subjects (58 males) were included in the study. The mean age of the study group was 8.98 ± 1.97 (SD) years old with an average FIQ of 112.79 ± 16.67 (SD).

**Table 1:** Subject demographics and characteristics.

Characteristics	Mean ± SD (n = 70)
Age	8.98 ± 1.97
Gender (female/male)	12/58
Full-scale IQ	112.79 ± 16.67
SRS Total	76.19 ± 16.15
ADI-R Social Interaction	18.57 ± 5.66
ADI-R Communication	15.14 ± 4.75
ADI-R RRB	5.77 ± 2.39

SRS, Social Responsiveness Scale; ADI-R, Autism Diagnostic Interview; RRB, Restrictive and Repetitive Behavior.

### Regions of Interest Identified From the Meta-Analysis

Twenty-one peak coordinates were identified from the uniformity test map of the meta-analysis (**Table 2**). The 21 coordinates were then used to create 6-mm radius spherical masks (see **Supplementary Material Figure S2** for the 21 spherical masks). The masks included the bilateral hippocampus/amygdala, bilateral fusiform gyrus, medial prefrontal cortex (mPFC), bilateral insula, bilateral ventral lateral prefrontal cortex (vlPFC), bilateral dorsal lateral prefrontal cortex (dlPFC), bilateral supplementary motor area (SMA), bilateral caudate, bilateral angular gyrus (AG), posterior cingulate gyrus (PCC), left middle temporal gyrus (MTG), left lateral occipital gyrus, right superior temporal gyrus (STG), left frontal eye field (FEF), left superior parietal gyrus, left postcentral gyrus, and left precentral gyrus. The masks were refined by taking the overlap of the masks and the original forward inference map. Then, the refined masks were used as ROIs in the seed-to-voxel connectivity analysis.

**Table 2:** Coordinates of ASD ROIs identified from meta-analysis.

Cluster ID	Cluster size	Peak T	Peak coordinates			Brain regions
			x	y	z	
1	390	11.3205	26	−6	−18	Hippocampus/amygdala_R (BA54/53)
2	355	11.3205	−22	−8	−20	Hippocampus/amygdala_L (BA54/53)
3	740	8.3575	40	−50	−20	Fusiform gyrus/LOC/MTG_R (BA37/19/21)
4	136	5.9871	−36	−50	−16	Fusiform gyrus_L (BA37)
5	533	7.1723	−2	58	20	mPFC (BA10)
6	86	7.1723	−60	−14	−14	aMTG_L (BA21)
7	1,053	10.1353	−34	22	4	Insula//IFG/dlPFC/precentral_L(BA13/44/45/47/9/6)
8	1,302	12.5057	36	22	0	Insula/IFG/dlPFC/precentral_R(BA13/44/45/47/9/6)
9	103	6.5797	22	−30	−4	Hippocampus_R (BA53)
10	52	5.9871	8	8	−4	Caudate_R (BA48)
11	271	8.3575	−46	−66	0	LOC_L (BA19)
12	65	5.3945	−10	10	−8	Caudate_L (BA48)
13	78	5.9871	−56	−42	2	pMTG/STS_L(BA21)
14	141	6.5797	54	−32	6	STS_R (BA22)
15	236	7.1723	50	−58	24	AG_R (BA39)
16	348	7.1723	−2	−54	24	PCC (BA23)
17	90	5.9871	−48	−66	28	AG_L (BA39)
18	397	8.3575	−2	10	52	SMA/FEF_L (BA6/8)
19	190	5.9871	−40	−42	48	SPL_L (BA7)
20	59	4.8019	−46	−30	52	Postcentral gyrus_L (BA1)
21	53	5.3945	−26	0	56	Precentral gyrus_L (BA6)

ASD, autism spectrum disorder; ROI, regions of interest; L, left; R, right; BA, Brodmann area; mPFC, medial prefrontal cortex; aMTG, anterior middle temporal gyrus; pMTG, posterior middle temporal gyrus; LOC, lateral occipital cortex; IFG, inferior frontal gyrus; dlPFC, dorsal lateral prefrontal; SMA, supplementary motor area; STS, superior temporal sulcus; AG, angular gyrus; PCC, posterior cingulate cortex; FEF, frontal eye field; SPL, superior parietal lobule.

### Potential NIBS Locations for ASD

**Figure 2:**
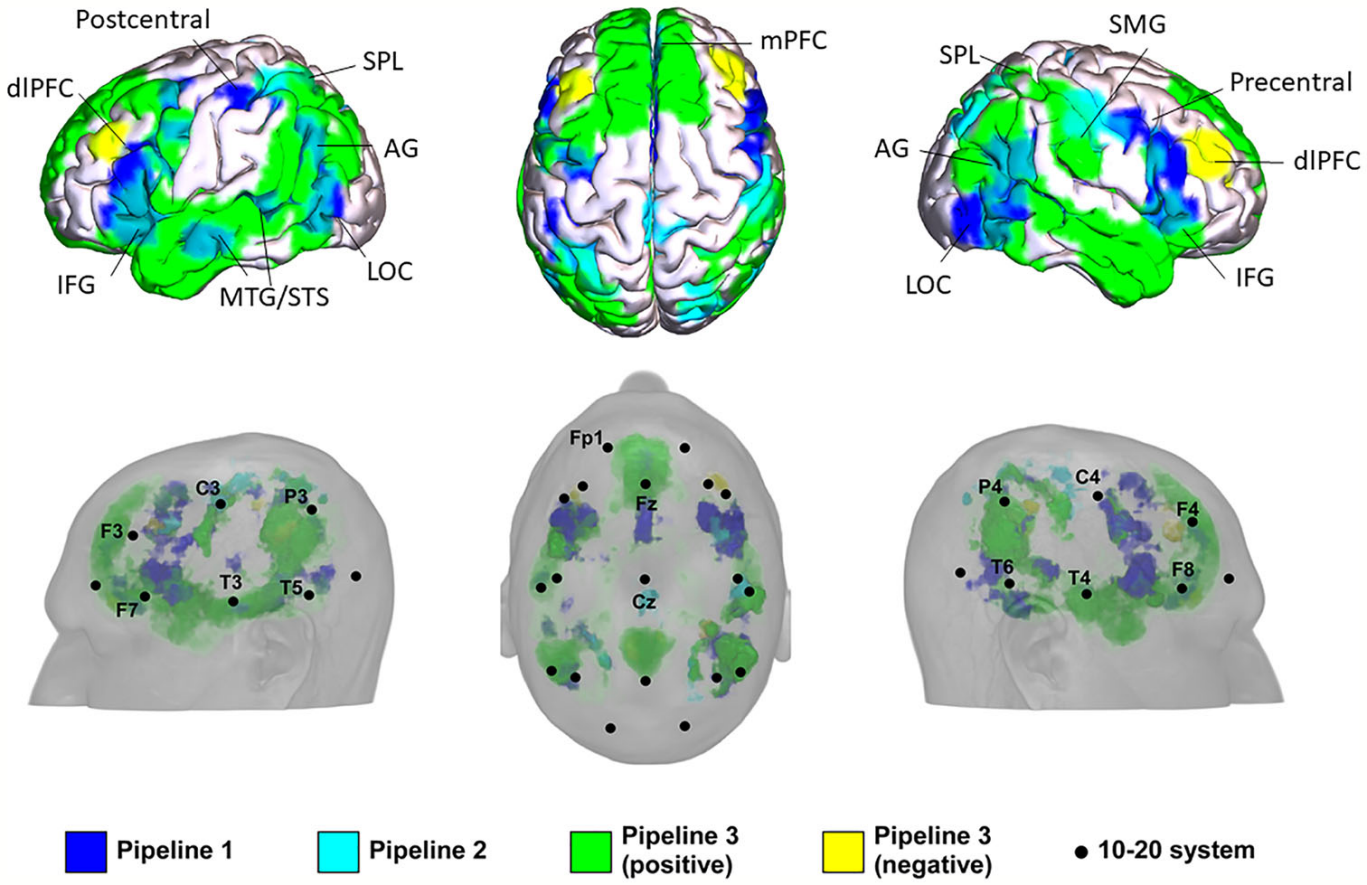
Potential locations for noninvasive brain stimulation (NIBS) in autism spectrum disorder (ASD) identified from the three pipelines. IFG, inferior frontal gyrus; AG, angular gyrus; LOC, lateral occipital cortex; mPFC, medial prefrontal cortex; STS, anterior superior temporal sulcus; MTG, middle temporal gyrus; dlPFC, dorsolateral prefrontal cortex; SMG, supramarginal gyrus; SPL, superior parietal lobule.

The results of the three pipelines were mapped onto a standard brain and a standard head in MNI space (**Table 3** and **Figure 2**).

**Table 3:** Potential locations for NIBS in ASD identified from the three pipelines.

Pipeline number		Identified brain regions	10-20 system locations
Pipeline 1		mPFC AG_bilateral dlPFC_bilateral IFG_bilateral Precentral_bilateral MTG/STS_bilateral LOC_bilateral SMA/FEF_L SPL_L Postcentral Gyrus_L	Fz P3 (P4) Posterior to F3 (F4) Posterior to F7 (F8) Anterior to C3 (C4) T3 (T4) T5 (T6) Posterior to Fz Anterior to P3 Posterior to C3
Pipeline 2	Positive	SPL_bilateral SMA/paracentral gyrus dlPFC_R AG_R Postcentral_R	Anterior to P3 (P4) Posterior to Cz Posterior to F4 P4 Posterior to C4
	Negative	None	None
Pipeline 3	Positive	mPFC Precuneus_bilateral TPO/MTG/STS_bilateral AG_bilateral dlPFC_bilateral IFG_bilateral SPL_bilateral Postcentral_bilateral LOC_bilateral Precentral_L SMG_R	Fz Posterior to Cz T3 (T4) P3 (P4) Posterior to F3 (F4) Posterior to F7 (F8) Anterior to P3 (P4) Posterior to C3 (C4) T5 (T6) Anterior to C3 Anterior to P4
	Negative	mPFC dlPFC_bilateral Precuneus_bilateral SMG_R	Fz Posterior to F3 (F4) Posterior to Cz Inferior to P4

positive: brain surface regions positively correlated with the ASD ROIs; negative: brain surface regions negatively correlated with the ASD ROIs.

L, left; R, right; mPFC, medial prefrontal cortex; AG, angular gyrus; IFG, inferior frontal gyrus; LOC, lateral occipital cortex; dlPFC, dorsal lateral prefrontal cortex; STS, superior temporal sulcus; TPO, temporal pole; MTG, middle temporal gyrus; SMA, supplementary motor area; FEF, frontal eye field; SPL, superior parietal lobule; ITG, inferior temporal gyrus; SMG, supramarginal gyrus.

In Pipeline 1, the mPFC, bilateral AG, bilateral dlPFC, inferior frontal gyrus (IFG), precentral gyrus, MTG/superior temporal sulcus (STS), lateral occipital cortex (LOC), left SMA/FEF, superior parietal lobe (SPL), and postcentral gyrus were identified as brain surface regions that may be directly involved in the pathophysiology of ASD (see **Supplementary Material Figure S3** for statistical maps of pipeline 1). The 10-20 system coordinates corresponding to the centers of these regions were located approximately at Fz, P3, and P4 (left and right AG), posterior to F3 and F4 (left and right dlPFC), posterior to F7 and F8 (left and right IFG), anterior to C3 and C4 (left and right precentral gyrus), center at T3 and T4 (left and right MTG), center at T5 and T6 (left and right LOC), posterior to Fz, anterior to P3, and posterior to C3.

In Pipeline 2, the bilateral SPL, SMA / paracentral gyrus, right dlPFC, AG, and postcentral gyrus were identified as brain surface regions positively correlated with the ASD network (see **Supplementary Material Figure S4** for statistical maps of pipeline 2). The 10-20 system coordinates corresponding to the centers of these regions were located approximately anterior to P3 and P4 (left and right SPL), posterior to Cz, posterior to F4, center at P4, and posterior to C4, respectively. No cluster was found to be negatively correlated with the ASD network.

In pipeline 3, the mPFC, bilateral precuneus, temporal pole (TPO)/MTG/STS, AG, dlPFC, IFG, SPL, postcentral gyrus, LOC, right supramarginal gyrus (SMG), and left precentral gyrus were identified as brain surface regions positively correlated with ASD ROIs (see **Supplementary Material Figure S5** for positive statistical maps of pipeline 3). The 10-20 system coordinates corresponding to the centers of these regions were located approximately at Fz, posterior to Cz, T3, and T4 (left and right TPO/MTG/STS), P3 and P4 (left and right AG), posterior to F3 and F4 (left and right dlPFC), posterior to F7 and F8 (left and right IFG), anterior to P3 and P4 (left and right SPL), posterior to C3 and C4 (left and right postcentral gyrus), center at T5 and T6 (left and right LOC), anterior to P4, and anterior to C3, respectively. The mPFC, bilateral dlPFC, precuneus, and right SMG were found to be brain surface regions negatively correlated with ASD ROIs (see **Supplementary Material Figure S6** for negative statistical maps of pipeline 3). The 10-20 system coordinates corresponding to the centers of these regions were located approximately at Fz, posterior to F3 and F4 (left and right dlPFC), posterior to Cz, and anterior to P4.

## Discussion

In this study, we combined resting-state functional connectivity and meta-analysis to identify potential NIBS targets for ASD. We found the brain regions overlapping between the meta-analysis and seed-based analysis to be potential targets, including the mPFC, bilateral AG, dlPFC, IFG, MTG/STS, LOC, left SPL, postcentral gyrus, precentral gyrus, and SMA. Stimulation locations on the scalp corresponding to these brain targets were also provided based on the EEG 10-20 system.

Consistent with the general consensus on cortical stimulation sites for ASD (10, 12), we identified the bilateral dlPFC, IFG, and AG as potential NIBS targets. The effectiveness of stimulating these three regions for ASD has been tested in previous studies (7, 30, 31).

Several neuropsychology studies have revealed impaired executive function in patients with pervasive developmental disorders such as ASD (32). Executive function encompasses a set of mental processes involved in planning, working memory, attention, problem solving, verbal reasoning, and mental flexibility (33), all of which are highly associated with activation of the dlPFC (34). As the most preferable target region, stimulation of the dlPFC may induce reduction in comorbid depression (35) and social-related impairments (36, 37) and yield improvement in attention (36) in individuals with ASD (38, 39). Sokhadze and colleagues reported improved executive functioning in individuals with ASD as evidence of normalization of event-related potential responses and behavioral accuracy after 1 Hz rTMS to the dlPFC (6). Thus, the dlPFC may be a potential target to treat symptoms associated with executive function and depression.

The IFG has been extensively associated with functions such as language processing, response inhibition, and social cognition and may be directly involved in the pathophysiology of ASD (40–46). For instance, Grace and her colleagues reported that activation in the left IFG was reduced in individuals with ASD compared to typically developing controls during speech stimulation (47). Moreover, Leehe et al. showed that anodal tDCS targeting the right IFG (rIFG) can modulate participants' emotional ratings to social touch.

As part of the TPJ, the AG integrates multi-sensory and cognitive processes that are implicated in the theory of mind and the attention network (48, 49). A recent study found that tDCS on the TPJ can influence social-cognitive performance as assessed by the Autism Spectrum Quotient (AQ) score (50). These studies demonstrate the functional alterations of the IFG and AG in ASD and provide support for using these regions as treatment targets, particularly in language and attention-related symptoms.

The motor system is another commonly used target for ASD (5). We found the left precentral gyrus and SMA in both the meta-analysis and seed-based analysis pipelines. A recent systematic review (11) on TMS neurophysiology revealed motor cortex excitatory and inhibitory imbalances in ASD, thereby providing a basis for targeting these regions. Thus, we speculate that the region may be associated with repetitive behavior and other motor-related symptoms.

In addition, our results suggest that the mPFC, bilateral MTG/STS, LOC, left SPL, and postcentral gyrus as other overlapping results of the meta-analysis and seed-based analysis should also be considered as potential targets for ASD treatment. Numerous functional imaging studies have demonstrated the important role of the prefrontal cortex in social cognition (51, 52), and the mPFC is particularly highlighted in the pathophysiology of ASD in both functional imaging and gene expression studies (53). Indeed, Enticott et al. found that deep rTMS to the bilateral mPFC can reduce social-related impairment and social-related anxiety in ASD patients (37), indicating that the mPFC may be a target region for these symptoms.

An extensive body of literature has shown the STS to be an established node of a “social network” (54) involved in language processing and social perception (55, 56), and it is a key region of numerous functional differences between ASD and typically developing individuals (57, 58). Beauchamp et al. reported that applying single-pulse TMS to the STS could significantly disrupt the McGurk effect (59), a perceptual phenomenon integrating hearing and vision in speech perception. Moreover, Daniel et al. found that theta burst TMS to the STS could produce a significant change in resting-state functional connectivity across the face-processing network (60). As findings have indicated that individuals with ASD may suffer from a weak McGurk effect and impaired face processing in daily settings (61, 62), these studies provide an interesting basis and guide for future stimulation of the STS in ASD treatment. Adjacent to the STG, the MTG is a brain area that is unique to humans (63). Previous studies have demonstrated the function of the MTG in language processing (64), and Acheson et al. reported a modulation effect on the language network when applying rTMS to the posterior MTG (64–67). These findings suggest that the STG and MTG may be potential targets for treating ASD symptoms related to language processing and social perception.

The SPL plays an important role in many cognitive, perceptive, and motor-related processes (68). In particular, event-related fMRI studies have shown that the SPL is critical for sensorimotor integration (69, 70), highlighting its potential role in the pathophysiology of repetitive and restrictive behaviors identified as additional core traits of ASD. Travers and colleagues reported that individuals with ASD had reduced fMRI activation in the SPL compared to typically developing individuals during motor-linked implicit learning, and they found that the more severe the traits of repetitive and restrictive behaviors, the greater the decrease in activation in the SPL (71). Furthermore, transcranial magnetic stimulation to the SPL was found to affect the planning of reaching movements (72). The SPL may therefore be considered a potential target for repetitive and restrictive behaviors.

As part of the lateral occipital-temporal complex (73), the LOC is an important region involved in object recognition (74) and multiple sensory integration. Studies have indicated that individuals with ASD may have difficulties integrating verbal and nonverbal cues during social interactions (75). Our previous study also found that reduced structural connectivity and resting-state brain activity in the LOC is associated with social communication deficits in boys with ASD (18).

Activation of the postcentral gyrus has been widely reported in previous studies when subjects were observing another person being touched (76, 77), suggesting the role of the postcentral gyrus in empathetic sharing of somatosensations. These findings suggest that this region may be used to relieve sensory-related symptoms in children with ASD (76, 77).

Finally, it is worth noting that identifying these locations may not necessarily be limited to NIBS, but may also be applied in other interventions such as scalp acupuncture (stimulating the area of scalp corresponding to brain regions believed to be involved in disorder pathology using acupuncture needles) and transcutaneous electrical nerve stimulation. Thus, results obtained from this study may facilitate the development of acupuncture and other therapeutic methods for the treatment of ASD.

There are several limitations to our study. First, the excitatory and inhibitory natures of these identified regions are indefinable using methods in this study. For some NIBS techniques like TMS, it is important to know the direction of the stimulation. How to apply and optimize different treatment modalities to target the brain areas identified in our study is beyond the scope of this manuscript. Investigators should consider the characteristics of different tools when attempting to stimulate these areas. Second, we do not know which pipeline is optimal for identifying potential NIBS targets for ASD. Regions identified from pipeline 1 are directly associated with ASD pathophysiology, regions identified from pipeline 2 have the strongest correlation with the ASD network, and regions identified from pipeline 3 correlate with the largest number of ASD ROIs. Understanding the derivative of these locations may help researchers choose target regions during clinical practice. Third, the meta-analysis conducted by Neurosynth is not flawless. Potential errors may occur during automatic extraction and synthesis of fMRI activation coordinates. However, several supporting analyses have been conducted to confirm the validity and sensitivity of Neurosynth-based meta-analysis and may provide evidence for the feasibility of this method. Finally, we applied peak z-scores to create spherical ROIs within these clusters. As a result, each ROI should have a similar size (voxel number), thus avoiding the potential influence of cluster size. Nevertheless, using original clusters as ROIs is also a reasonable method. One potential limitation of using the cluster derived from Neurosynth is that the cluster size may vary when different thresholds are applied.

In conclusion, we identified several potential NIBS targets and their corresponding stimulation locations on the scalp for the treatment of ASD. As ASD displays significant clinical heterogeneity with respect to stimulation sites, a clear link between neurobiological targets and clinical outcome measurements may be a future step toward optimizing NIBS for ASD treatment. Although further testing of these identified targets is needed, these results may help clinicians optimize the application of NIBS therapy in individuals with ASD.

## Data Availability Statement

Publicly available datasets were analyzed in this study. This data can be found at: http://fcon_1000.projects.nitrc.org/indi/abide/abide_II.html.

## Ethics Statement

The study used public datasets from 3 centers. Please refer to http://fcon_1000.projects.nitrc.org/indi/abide/abide_II.html for details. Written informed consent to participate in this study was provided by the participants' legal guardian/next of kin.

## Author Contributions

YH contributed to data analysis and manuscript preparation. JK conceived the idea, contributed to data analysis and manuscript preparation, BZ contributed to data analysis and manuscript preparation, contributed to study design and manuscript preparation. JC contributed to manuscript revision. SY contributed to data analysis. GW and JP contributed to manuscript preparation.

## Conflict of Interest

JK holds equity in a startup company, MNT, and has pending patents to develop new neuromodulation tools.

The remaining authors declare that the research was conducted in the absence of any commercial or financial relationships that could be construed as a potential conflict of interest.
