# Supplementary material for: Potential Locations for Noninvasive Brain Stimulation in Treating Autism Spectrum Disorders—A Functional Connectivity Study
Source: Front Psychiatry. 2020 May 7;11:388. doi: 10.3389/fpsyt.2020.00388 (PMC7221195; doi:10.3389/fpsyt.2020.00388)
Supplement: Supplementary file 1 [file DataSheet_1.docx]

**Supplementary materials for:**

**Potential locations for noninvasive brain stimulation in treating**

**autism spectrum disorders – a functional connectivity study**

Yiting Huang, ^1#^Binlong Zhang^1#^, Jin Cao^1^, Siyi Yu^1^, Georgia Wilson^1^, Joel Park^1^, Jian Kong^1*^

^1^Department of Psychiatry, Massachusetts General Hospital, Harvard Medical School, Boston, MA, USA

^#^These authors contributed equally to this work.

**^*^Correspondence to:**

Jian Kong

Department of Psychiatry

Massachusetts General Hospital

120 2^nd^ Ave, Room 101C, Charlestown, MA 02129

Phone: +1 617-286-9879

Fax: +1 617-643-7340

E-mail: jkong2@mgh.harvard.edu


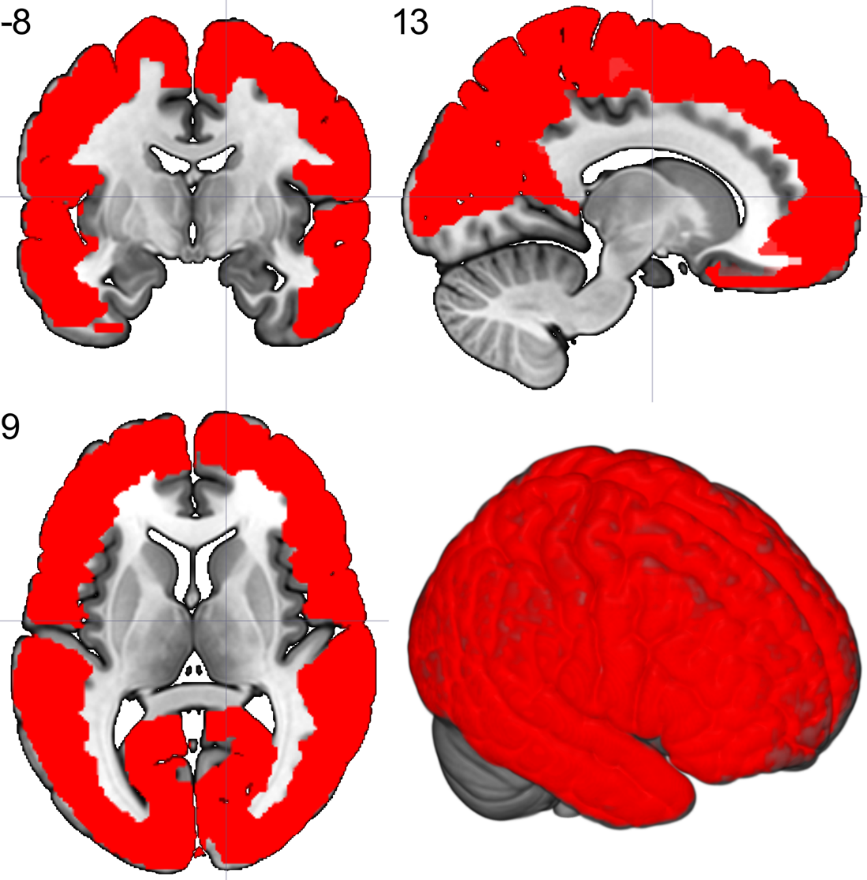


**Fig. S1**. Brain surface mask used to exclude brain regions not on the brain surface after one-sample t-test functional connectivity analysis.


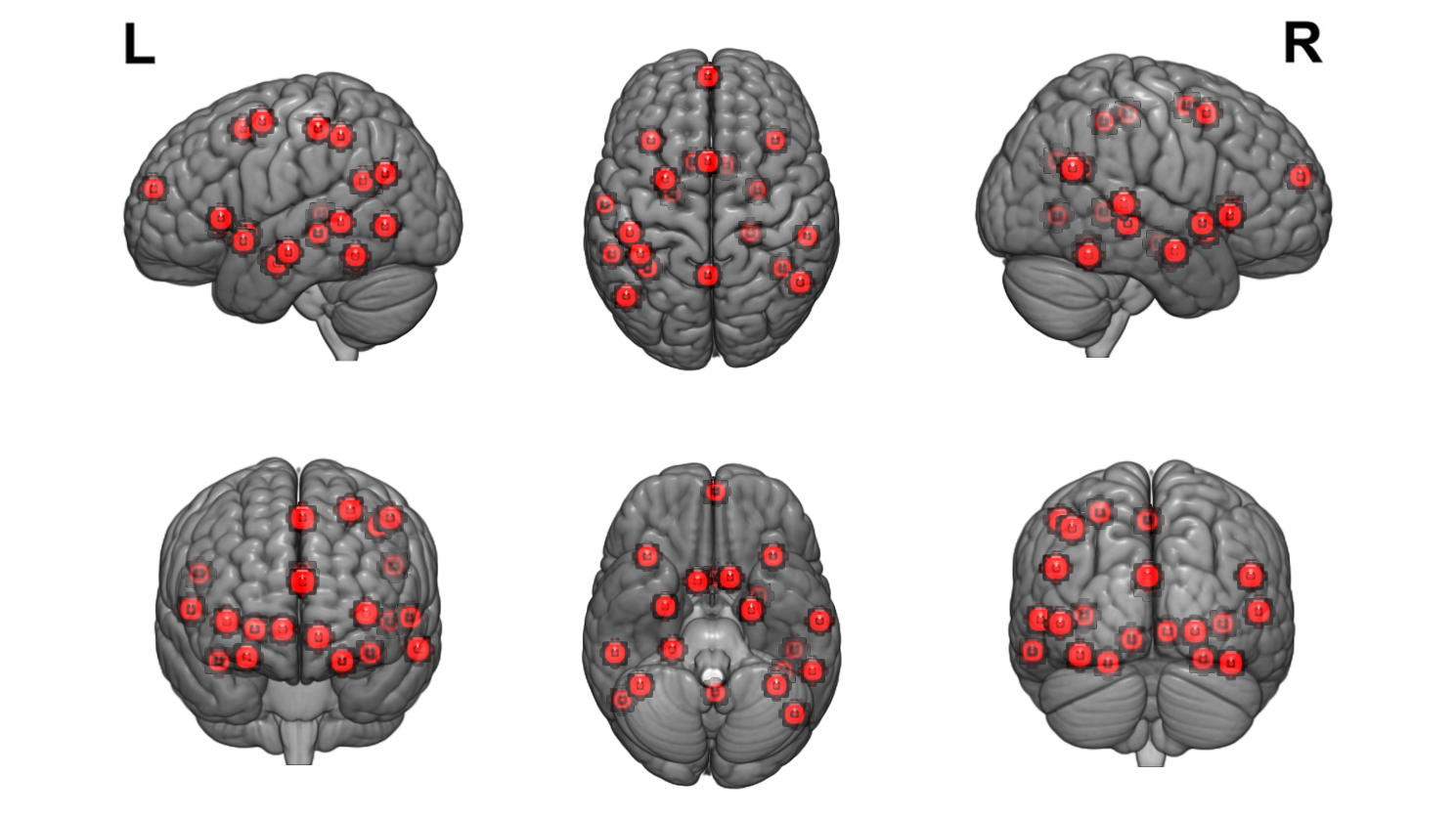


**Fig. S2**. The 6-mm radius spherical masks created from 21 peak coordinates (spherical masks were further refined by taking the overlap of the uniformity test map)

**
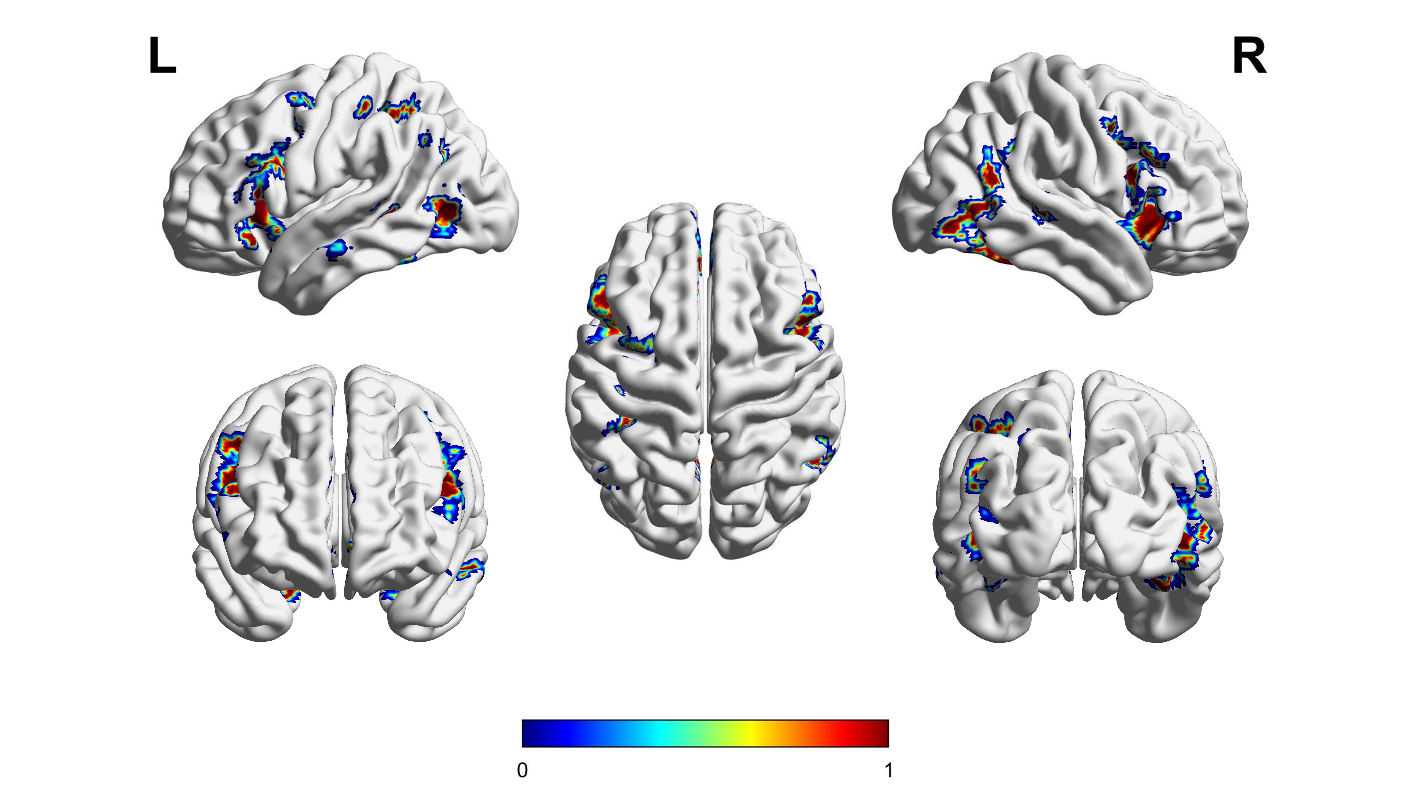
**

**Fig. S3**. Results of Pipeline 1. Mapping the uniform test map on the brain surface (cluster size>50)


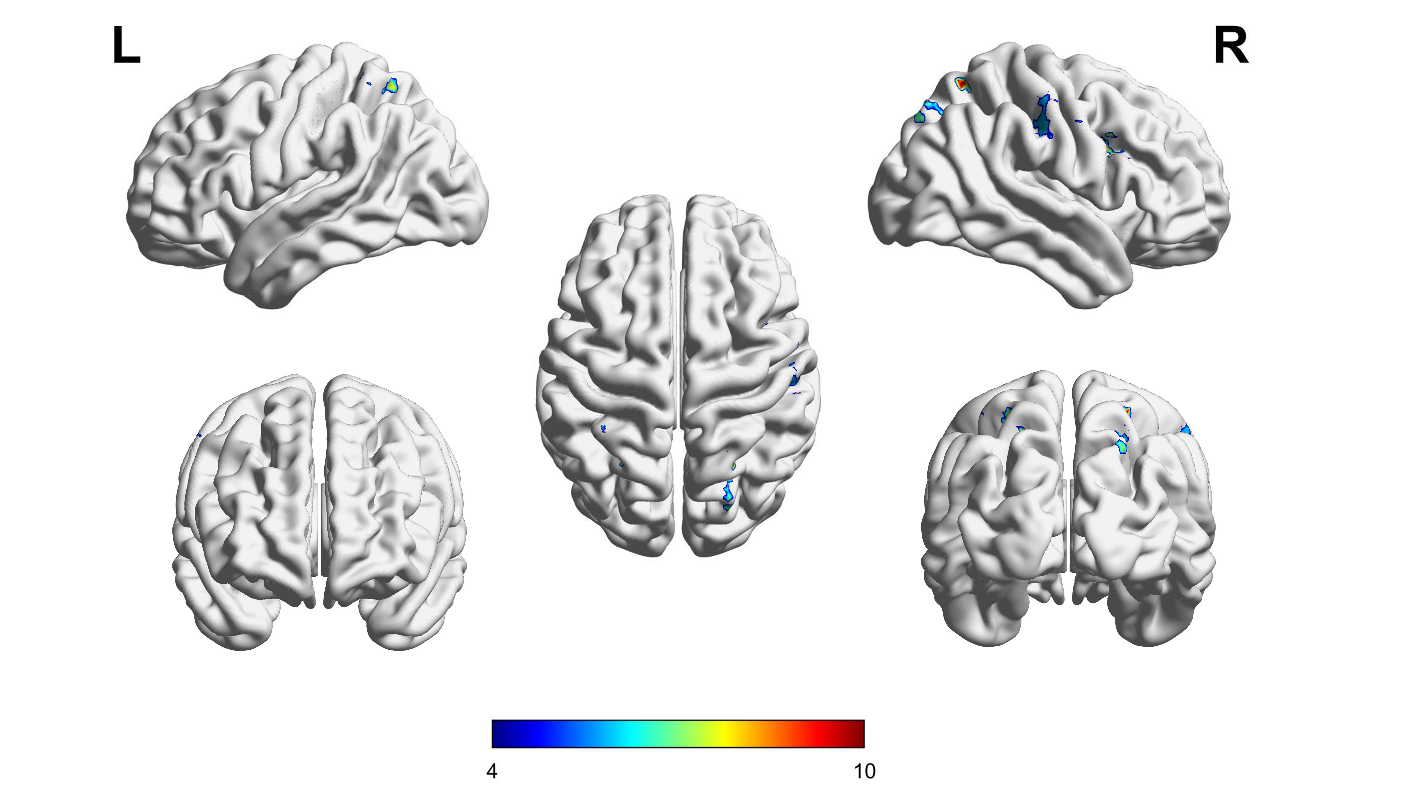


**Fig. S4**. Results of Pipeline 2 (positive). Group-level correlation (positive) map of the autism spectrum disorder network (cluster size>50)

**
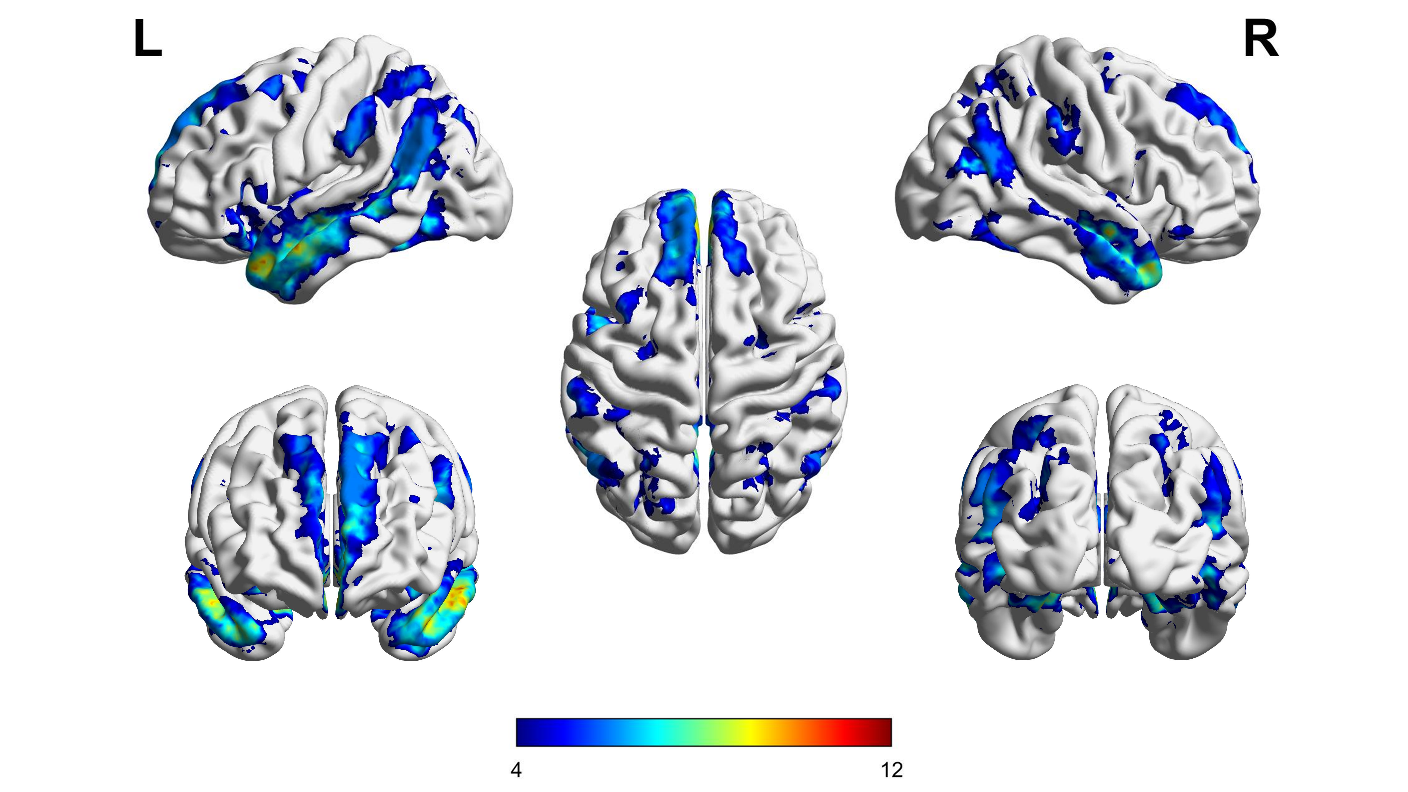
**

**Fig. S5**. Results of Pipeline 3 (positive). Third-level correlation map (positive) of autism spectrum disorder associated ROIs (the intensity of each voxel in the map represents the number of ROIs correlated to the voxel)


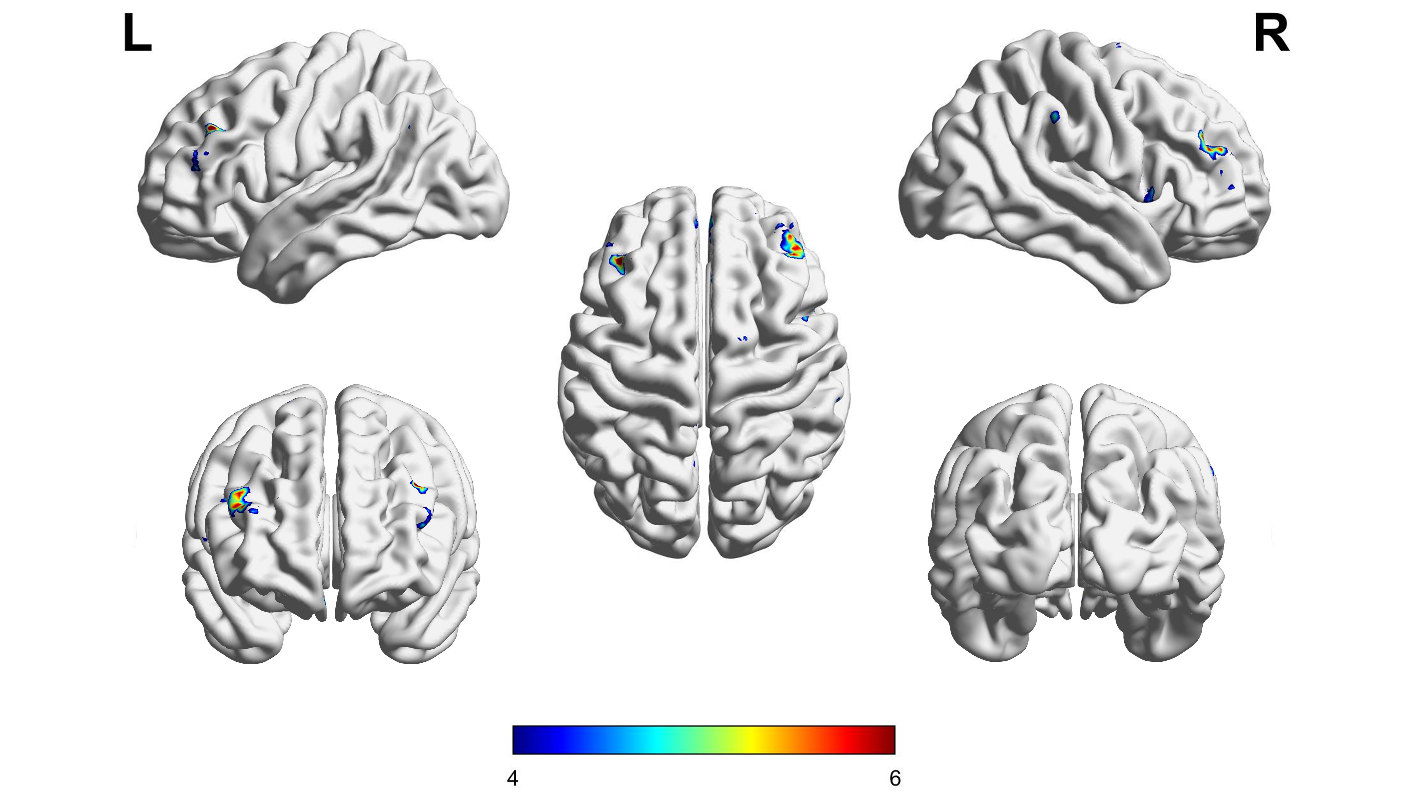


**Fig. S6**. Results of Pipeline 3 (negative). Third-level correlation map (negative) of autism spectrum disorder associated ROIs (the intensity of each voxel in the map represents the number of ROIs correlated to the voxel)

**Table S1.** List of 170 studies extracted from Neurosynth under search term of “autism spectrum” on March 11, 2020

|  | **Year** | **Title** | **Authors** | **Journal** |
| --- | --- | --- | --- | --- |
| **1** | **2003** | Functional anatomy of impaired selective attention and compensatory processing in autism. | Belmonte MK, Yurgelun-Todd DA | Brain Research: Cognitive Brain Research |
| **2** | **2004** | The neural basis of hyperlexic reading: an FMRI case study. | Turkeltaub PE, Flowers DL, Verbalis A, Miranda M, Gareau L, Eden GF | Neuron |
| **3** | **2005** | Mapping the brain in autism. A voxel-based MRI study of volumetric differences and intercorrelations in autism. | McAlonan GM, Cheung V, Cheung C, Suckling J, Lam GY, Tai KS, Yip L, Murphy DG, Chua SE | Brain: A Journal of Neurology |
| **4** | **2006** | Brain activation during semantic processing in autism spectrum disorders via functional magnetic resonance imaging. | Harris GJ, Chabris CF, Clark J, Urban T, Aharon I, Steele S, McGrath L, Condouris K, Tager-Flusberg H | Brain and Cognition |
| **5** | **2006** | Neural basis of irony comprehension in children with autism: the role of prosody and context. | Wang AT, Lee SS, Sigman M, Dapretto M | Brain: A Journal of Neurology |
| **6** | **2007** | Histological and magnetic resonance imaging assessment of cortical layering and thickness in autism spectrum disorders. | Hutsler JJ, Love T, Zhang H | Biological Psychiatry |
| **7** | **2007** | Atypical [corrected] participation of visual cortex during word processing in autism: an fMRI study of semantic decision. | Gaffrey MS, Kleinhans NM, Haist F, Akshoomoff N, Campbell A, Courchesne E, Muller RA | Neuropsychologia |
| **8** | **2007** | Atypical neural substrates of Embedded Figures Task performance in children with Autism Spectrum Disorder. | Lee PS, Foss-Feig J, Henderson JG, Kenworthy LE, Gilotty L, Gaillard WD, Vaidya CJ | NeuroImage |
| **9** | **2007** | Amygdala dysfunction in men with the fragile X premutation. | Hessl D, Rivera S, Koldewyn K, Cordeiro L, Adams J, Tassone F, Hagerman PJ, Hagerman RJ | Brain: A Journal of Neurology |
| **10** | **2007** | Social stimuli interfere with cognitive control in autism. | Dichter GS, Belger A | NeuroImage |
| **11** | **2008** | Neural bases for impaired social cognition in schizophrenia and autism spectrum disorders. | Pinkham AE, Hopfinger JB, Pelphrey KA, Piven J, Penn DL | Schizophrenia Research |
| **12** | **2008** | Atypical functional lateralization of language in autism spectrum disorders. | Kleinhans NM, Muller RA, Cohen DN, Courchesne E | Brain Research |
| **13** | **2008** | Deviant functional magnetic resonance imaging patterns of brain activity to speech in 2-3-year-old children with autism spectrum disorder. | Redcay E, Courchesne E | Biological Psychiatry |
| **14** | **2008** | Atypical recruitment of medial prefrontal cortex in autism spectrum disorders: an fMRI study of two executive function tasks. | Gilbert SJ, Bird G, Brindley R, Frith CD, Burgess PW | Neuropsychologia |
| **15** | **2008** | Neural basis of self and other representation in autism: an FMRI study of self-face recognition. | Uddin LQ, Davies MS, Scott AA, Zaidel E, Bookheimer SY, Iacoboni M, Dapretto M | PloS One |
| **16** | **2008** | Atypical modulation of cognitive control by arousal in autism. | Dichter GS, Belger A | Psychiatry Research |
| **17** | **2008** | Atypical modulation of distant functional connectivity by cognitive state in children with Autism Spectrum Disorders. | You X, Norr M, Murphy E, Kuschner ES, Bal E, Gaillard WD, Kenworthy L, Vaidya CJ | Frontiers in Human Neuroscience |
| **18** | **2008** | Perception of biological motion in autism spectrum disorders. | Freitag CM, Konrad C, Haberlen M, Kleser C, von Gontard A, Reith W, Troje NF, Krick C | Neuropsychologia |
| **19** | **2008** | Response monitoring, repetitive behaviour and anterior cingulate abnormalities in autism spectrum disorders (ASD). | Thakkar KN, Polli FE, Joseph RM, Tuch DS, Hadjikhani N, Barton JJ, Manoach DS | Brain: A Journal of Neurology |
| **20** | **2009** | The neural substrates of cognitive control deficits in autism spectrum disorders. | Solomon M, Ozonoff SJ, Ursu S, Ravizza S, Cummings N, Ly S, Carter CS | Neuropsychologia |
| **21** | **2009** | Body expressions of emotion do not trigger fear contagion in autism spectrum disorder. | Hadjikhani N, Joseph RM, Manoach DS, Naik P, Snyder J, Dominick K, Hoge R, Van den Stock J, Tager Flusberg H, de Gelder B | Social Cognitive and Affective Neuroscience |
| **22** | **2009** | Aberrant functional connectivity in autism: evidence from low-frequency BOLD signal fluctuations. | Noonan SK, Haist F, Muller RA | Brain Research |
| **23** | **2009** | Functional connectivity of the inferior frontal cortex changes with age in children with autism spectrum disorders: a fcMRI study of response inhibition. | Lee PS, Yerys BE, Della Rosa A, Foss-Feig J, Barnes KA, James JD, VanMeter J, Vaidya CJ, Gaillard WD, Kenworthy LE | Cerebral Cortex (New York, NY, 1991) |
| **24** | **2009** | Autism is characterized by dorsal anterior cingulate hyperactivation during social target detection. | Dichter GS, Felder JN, Bodfish JW | Social Cognitive and Affective Neuroscience |
| **25** | **2009** | Neural correlates of pragmatic language comprehension in autism spectrum disorders. | Tesink CM, Buitelaar JK, Petersson KM, van der Gaag RJ, Kan CC, Tendolkar I, Hagoort P | Brain: A Journal of Neurology |
| **26** | **2009** | Abnormalities of intrinsic functional connectivity in autism spectrum disorders. | Monk CS, Peltier SJ, Wiggins JL, Weng SJ, Carrasco M, Risi S, Lord C | NeuroImage |
| **27** | **2010** | Atypical activation of the mirror neuron system during perception of hand motion in autism. | Martineau J, Andersson F, Barthelemy C, Cottier JP, Destrieux C | Brain Research |
| **28** | **2010** | Neural signatures of autism. | Kaiser MD, Hudac CM, Shultz S, Lee SM, Cheung C, Berken AM, Deen B, Pitskel NB, Sugrue DR, Voos AC, Saulnier CA, Ventola P, Wolf JM, Klin A, Vander Wyk BC, Pelphrey KA | Proceedings of the National Academy of Sciences of the United States of America |
| **29** | **2010** | Amygdala engagement in response to subthreshold presentations of anxious face stimuli in adults with autism spectrum disorders: preliminary insights. | Hall GB, Doyle KA, Goldberg J, West D, Szatmari P | PloS One |
| **30** | **2010** | Sources of group differences in functional connectivity: an investigation applied to autism spectrum disorder. | Jones TB, Bandettini PA, Kenworthy L, Case LK, Milleville SC, Martin A, Birn RM | NeuroImage |
| **31** | **2010** | Association between amygdala response to emotional faces and social anxiety in autism spectrum disorders. | Kleinhans NM, Richards T, Weaver K, Johnson LC, Greenson J, Dawson G, Aylward E | Neuropsychologia |
| **32** | **2010** | Regional homogeneity of fMRI time series in autism spectrum disorders. | Shukla DK, Keehn B, Muller RA | Neuroscience Letters |
| **33** | **2010** | Abnormal functional connectivity of default mode sub-networks in autism spectrum disorder patients. | Assaf M, Jagannathan K, Calhoun VD, Miller L, Stevens MC, Sahl R, O'Boyle JG, Schultz RT, Pearlson GD | NeuroImage |
| **34** | **2010** | Describing the brain in autism in five dimensions--magnetic resonance imaging-assisted diagnosis of autism spectrum disorder using a multiparameter classification approach. | Ecker C, Marquand A, Mourao-Miranda J, Johnston P, Daly EM, Brammer MJ, Maltezos S, Murphy CM, Robertson D, Williams SC, Murphy DG | The Journal of Neuroscience: The Official Journal of the Society for Neuroscience |
| **35** | **2010** | Alterations in regional homogeneity of resting-state brain activity in autism spectrum disorders. | Paakki JJ, Rahko J, Long X, Moilanen I, Tervonen O, Nikkinen J, Starck T, Remes J, Hurtig T, Haapsamo H, Jussila K, Kuusikko-Gauffin S, Mattila ML, Zang Y, Kiviniemi V | Brain Research |
| **36** | **2010** | Coherent motion processing in autism spectrum disorder (ASD): an fMRI study. | Brieber S, Herpertz-Dahlmann B, Fink GR, Kamp-Becker I, Remschmidt H, Konrad K | Neuropsychologia |
| **37** | **2010** | Reduced cognitive control of response inhibition by the anterior cingulate cortex in autism spectrum disorders. | Agam Y, Joseph RM, Barton JJ, Manoach DS | NeuroImage |
| **38** | **2010** | No neural evidence of statistical learning during exposure to artificial languages in children with autism spectrum disorders. | Scott-Van Zeeland AA, McNealy K, Wang AT, Sigman M, Bookheimer SY, Dapretto M | Biological Psychiatry |
| **39** | **2010** | Smaller insula and inferior frontal volumes in young adults with pervasive developmental disorders. | Kosaka H, Omori M, Munesue T, Ishitobi M, Matsumura Y, Takahashi T, Narita K, Murata T, Saito DN, Uchiyama H, Morita T, Kikuchi M, Mizukami K, Okazawa H, Sadato N, Wada Y | NeuroImage |
| **40** | **2010** | Alterations of resting state functional connectivity in the default network in adolescents with autism spectrum disorders. | Weng SJ, Wiggins JL, Peltier SJ, Carrasco M, Risi S, Lord C, Monk CS | Brain Research |
| **41** | **2010** | Atypical neural self-representation in autism. | Lombardo MV, Chakrabarti B, Bullmore ET, Sadek SA, Pasco G, Wheelwright SJ, Suckling J, Baron-Cohen S | Brain: A Journal of Neurology |
| **42** | **2011** | Autism spectrum traits in the typical population predict structure and function in the posterior superior temporal sulcus. | von dem Hagen EA, Nummenmaa L, Yu R, Engell AD, Ewbank MP, Calder AJ | Cerebral Cortex (New York, NY, 1991) |
| **43** | **2011** | Discrete neural substrates underlie complementary audiovisual speech integration processes. | Stevenson RA, VanDerKlok RM, Pisoni DB, James TW | NeuroImage |
| **44** | **2011** | Sex-linked white matter microstructure of the social and analytic brain. | Chou KH, Cheng Y, Chen IY, Lin CP, Chu WC | NeuroImage |
| **45** | **2011** | Multivariate searchlight classification of structural magnetic resonance imaging in children and adolescents with autism. | Uddin LQ, Menon V, Young CB, Ryali S, Chen T, Khouzam A, Minshew NJ, Hardan AY | Biological Psychiatry |
| **46** | **2011** | Feature selection and classification of imbalanced datasets: application to PET images of children with autistic spectrum disorders. | Duchesnay E, Cachia A, Boddaert N, Chabane N, Mangin JF, Martinot JL, Brunelle F, Zilbovicius M | NeuroImage |
| **47** | **2011** | Functional differentiation of posterior superior temporal sulcus in autism: a functional connectivity magnetic resonance imaging study. | Shih P, Keehn B, Oram JK, Leyden KM, Keown CL, Muller RA | Biological Psychiatry |
| **48** | **2011** | Using a self-organizing map algorithm to detect age-related changes in functional connectivity during rest in autism spectrum disorders. | Wiggins JL, Peltier SJ, Ashinoff S, Weng SJ, Carrasco M, Welsh RC, Lord C, Monk CS | Brain Research |
| **49** | **2011** | Aberrant striatal functional connectivity in children with autism. | Di Martino A, Kelly C, Grzadzinski R, Zuo XN, Mennes M, Mairena MA, Lord C, Castellanos FX, Milham MP | Biological Psychiatry |
| **50** | **2011** | Atypical neural networks for social orienting in autism spectrum disorders. | Greene DJ, Colich N, Iacoboni M, Zaidel E, Bookheimer SY, Dapretto M | NeuroImage |
| **51** | **2011** | Neural bases of gaze and emotion processing in children with autism spectrum disorders. | Davies MS, Dapretto M, Sigman M, Sepeta L, Bookheimer SY | Brain and Behavior |
| **52** | **2012** | Fractionation of social brain circuits in autism spectrum disorders. | Gotts SJ, Simmons WK, Milbury LA, Wallace GL, Cox RW, Martin A | Brain: A Journal of Neurology |
| **53** | **2012** | Autism spectrum traits predict the neural response to eye gaze in typical individuals. | Nummenmaa L, Engell AD, von dem Hagen E, Henson RN, Calder AJ | NeuroImage |
| **54** | **2012** | Functional alterations in neural substrates of geometric reasoning in adults with high-functioning autism. | Yamada T, Ohta H, Watanabe H, Kanai C, Tani M, Ohno T, Takayama Y, Iwanami A, Kato N, Hashimoto R | PloS one |
| **55** | **2012** | Do distinct atypical cortical networks process biological motion information in adults with Autism Spectrum Disorders? | McKay LS, Simmons DR, McAleer P, Marjoram D, Piggot J, Pollick FE | NeuroImage |
| **56** | **2012** | DNA methylation of the oxytocin receptor gene predicts neural response to ambiguous social stimuli. | Jack A, Connelly JJ, Morris JP | Frontiers in Human Neuroscience |
| **57** | **2012** | Reward circuitry function in autism during face anticipation and outcomes. | Dichter GS, Richey JA, Rittenberg AM, Sabatino A, Bodfish JW | Journal of Autism and Developmental Disorders |
| **58** | **2012** | Reduced functional integration and segregation of distributed neural systems underlying social and emotional information processing in autism spectrum disorders. | Rudie JD, Shehzad Z, Hernandez LM, Colich NL, Bookheimer SY, Iacoboni M, Dapretto M | Cerebral Cortex (New York, NY, 1991) |
| **59** | **2012** | An fMRI study of reduced perceptual load-dependent modulation of task-irrelevant activity in adults with autism spectrum conditions. | Ohta H, Yamada T, Watanabe H, Kanai C, Tanaka E, Ohno T, Takayama Y, Iwanami A, Kato N, Hashimoto R | NeuroImage |
| **60** | **2012** | Female children with autism spectrum disorder: an insight from mass-univariate and pattern classification analyses. | Calderoni S, Retico A, Biagi L, Tancredi R, Muratori F, Tosetti M | NeuroImage |
| **61** | **2012** | The superior temporal sulcus differentiates communicative and noncommunicative auditory signals. | Shultz S, Vouloumanos A, Pelphrey K | Journal of Cognitive Neuroscience |
| **62** | **2012** | Sex differences and autism: brain function during verbal fluency and mental rotation. | Beacher FD, Radulescu E, Minati L, Baron-Cohen S, Lombardo MV, Lai MC, Walker A, Howard D, Gray MA, Harrison NA, Critchley HD | PloS One |
| **63** | **2012** | Functional deficits of the attentional networks in autism. | Fan J, Bernardi S, Van Dam NT, Anagnostou E, Gu X, Martin L, Park Y, Liu X, Kolevzon A, Soorya L, Grodberg D, Hollander E, Hof PR | Brain and Behavior |
| **64** | **2012** | Reward circuitry function in autism spectrum disorders. | Dichter GS, Felder JN, Green SR, Rittenberg AM, Sasson NJ, Bodfish JW | Social Cognitive and Affective Neuroscience |
| **65** | **2012** | Diminished medial prefrontal activity behind autistic social judgments of incongruent information. | Watanabe T, Yahata N, Abe O, Kuwabara H, Inoue H, Takano Y, Iwashiro N, Natsubori T, Aoki Y, Takao H, Sasaki H, Gonoi W, Murakami M, Katsura M, Kunimatsu A, Kawakubo Y, Matsuzaki H, Tsuchiya KJ, Kato N, Kano Y, Miyashita Y, Kasai K, Yamasue H | PloS One |
| **66** | **2012** | Social cognition, the male brain and the autism spectrum. | Hall J, Philip RC, Marwick K, Whalley HC, Romaniuk L, McIntosh AM, Santos I, Sprengelmeyer R, Johnstone EC, Stanfield AC, Young AW, Lawrie SM | PloS One |
| **67** | **2012** | Atypical lexicosemantic function of extrastriate cortex in autism spectrum disorder: evidence from functional and effective connectivity. | Shen MD, Shih P, Ottl B, Keehn B, Leyden KM, Gaffrey MS, Muller RA | NeuroImage |
| **68** | **2012** | Age-related abnormalities in white matter microstructure in autism spectrum disorders. | Kleinhans NM, Pauley G, Richards T, Neuhaus E, Martin N, Corrigan NM, Shaw DW, Estes A, Dager SR | Brain Research |
| **69** | **2012** | Altered integration of speech and gesture in children with autism spectrum disorders. | Hubbard AL, McNealy K, Scott-Van Zeeland AA, Callan DE, Bookheimer SY, Dapretto M | Brain and Behavior |
| **70** | **2012** | The role of the amygdala in atypical gaze on emotional faces in autism spectrum disorders. | Kliemann D, Dziobek I, Hatri A, Baudewig J, Heekeren HR | The Journal of Neuroscience: The Official Journal of the Society for Neuroscience |
| **71** | **2012** | The impact of serotonin transporter (5-HTTLPR) genotype on the development of resting-state functional connectivity in children and adolescents: a preliminary report. | Wiggins JL, Bedoyan JK, Peltier SJ, Ashinoff S, Carrasco M, Weng SJ, Welsh RC, Martin DM, Monk CS | NeuroImage |
| **72** | **2012** | Hard to "tune in": neural mechanisms of live face-to-face interaction with high-functioning autistic spectrum disorder. | Tanabe HC, Kosaka H, Saito DN, Koike T, Hayashi MJ, Izuma K, Komeda H, Ishitobi M, Omori M, Munesue T, Okazawa H, Wada Y, Sadato N | Frontiers in Human Neuroscience |
| **73** | **2013** | Evaluation of enhanced attention to local detail in anorexia nervosa using the embedded figures test; an FMRI study. | Fonville L, Lao-Kaim NP, Giampietro V, Van den Eynde F, Davies H, Lounes N, Andrew C, Dalton J, Simmons A, Williams SC, Baron-Cohen S, Tchanturia K | PloS One |
| **74** | **2013** | Neural and behavioral responses during self-evaluative processes differ in youth with and without autism. | Pfeifer JH, Merchant JS, Colich NL, Hernandez LM, Rudie JD, Dapretto M | Journal of Autism and Developmental Disorders |
| **75** | **2013** | Effects of intranasal oxytocin on the neural basis of face processing in autism spectrum disorder. | Domes G, Heinrichs M, Kumbier E, Grossmann A, Hauenstein K, Herpertz SC | Biological Psychiatry |
| **76** | **2013** | Oxytocin enhances brain function in children with autism. | Gordon I, Vander Wyk BC, Bennett RH, Cordeaux C, Lucas MV, Eilbott JA, Zagoory-Sharon O, Leckman JF, Feldman R, Pelphrey KA | Proceedings of the National Academy of Sciences of the United States of America |
| **77** | **2013** | Neural correlate of autistic-like traits and a common allele in the oxytocin receptor gene. | Saito Y, Suga M, Tochigi M, Abe O, Yahata N, Kawakubo Y, Liu X, Kawamura Y, Sasaki T, Kasai K, Yamasue H | Social Cognitive and Affective Neuroscience |
| **78** | **2013** | Neural correlates of moral reasoning in autism spectrum disorder. | Schneider K, Pauly KD, Gossen A, Mevissen L, Michel TM, Gur RC, Schneider F, Habel U | Social Cognitive and Affective Neuroscience |
| **79** | **2013** | Approaches to local connectivity in autism using resting state functional connectivity MRI. | Maximo JO, Keown CL, Nair A, Muller RA | Frontiers in Human Neuroscience |
| **80** | **2013** | It's all in the eyes: subcortical and cortical activation during grotesqueness perception in autism. | Zurcher NR, Donnelly N, Rogier O, Russo B, Hippolyte L, Hadwin J, Lemonnier E, Hadjikhani N | PloS One |
| **81** | **2013** | Reduced functional connectivity within and between 'social' resting state networks in autism spectrum conditions. | von dem Hagen EA, Stoyanova RS, Baron-Cohen S, Calder AJ | Social Cognitive and Affective Neuroscience |
| **82** | **2013** | Neural correlates of inhibition of socially relevant stimuli in adults with autism spectrum disorder. | Duerden EG, Taylor MJ, Soorya LV, Wang T, Fan J, Anagnostou E | Brain Research |
| **83** | **2013** | Age related changes in striatal resting state functional connectivity in autism. | Padmanabhan A, Lynn A, Foran W, Luna B, O'Hearn K | Frontiers in Human Neuroscience |
| **84** | **2013** | Age-dependent changes in the neural substrates of empathy in autism spectrum disorder. | Schulte-Ruther M, Greimel E, Piefke M, Kamp-Becker I, Remschmidt H, Fink GR, Herpertz-Dahlmann B, Konrad K | Social Cognitive and Affective Neuroscience |
| **85** | **2013** | Functional connectivity for an "island of sparing" in autism spectrum disorder: an fMRI study of visual search. | Keehn B, Shih P, Brenner LA, Townsend J, Muller RA | Human Brain Mapping |
| **86** | **2013** | Perception of social cues of danger in autism spectrum disorders. | Zurcher NR, Rogier O, Boshyan J, Hippolyte L, Russo B, Gillberg N, Helles A, Ruest T, Lemonnier E, Gillberg C, Hadjikhani N | PloS One |
| **87** | **2013** | Phonological processing in first-degree relatives of individuals with autism: an fMRI study. | Wilson LB, Tregellas JR, Slason E, Pasko BE, Hepburn S, Rojas DC | Human Brain Mapping |
| **88** | **2013** | Convergent Findings of Altered Functional and Structural Brain Connectivity in Individuals with High Functioning Autism: A Multimodal MRI Study. | Mueller S, Keeser D, Samson AC, Kirsch V, Blautzik J, Grothe M, Erat O, Hegenloh M, Coates U, Reiser MF, Hennig-Fast K, Meindl T | PloS One |
| **89** | **2013** | Differences in global and local level information processing in autism: an fMRI investigation. | Gadgil M, Peterson E, Tregellas J, Hepburn S, Rojas DC | Psychiatry Research |
| **90** | **2013** | Reward system dysfunction in autism spectrum disorders. | Kohls G, Schulte-Ruther M, Nehrkorn B, Muller K, Fink GR, Kamp-Becker I, Herpertz-Dahlmann B, Schultz RT, Konrad K | Social Cognitive and Affective Neuroscience |
| **91** | **2013** | Aberrant neural mediation of verbal fluency in autism spectrum disorders. | Kenworthy L, Wallace GL, Birn R, Milleville SC, Case LK, Bandettini PA, Martin A | Brain and Cognition |
| **92** | **2013** | The Development of the Neural Substrates of Cognitive Control in Adolescents with Autism Spectrum Disorders. | Solomon M, Yoon JH, Ragland JD, Niendam TA, Lesh TA, Fairbrother W, Carter CS | Biological Psychiatry |
| **93** | **2013** | Structural and functional underconnectivity as a negative predictor for language in autism. | Verly M, Verhoeven J, Zink I, Mantini D, Oudenhove LV, Lagae L, Sunaert S, Rommel N | Human Brain Mapping |
| **94** | **2013** | Atypical brain activation patterns during a face-to-face joint attention game in adults with autism spectrum disorder. | Redcay E, Dodell-Feder D, Mavros PL, Kleiner M, Pearrow MJ, Triantafyllou C, Gabrieli JD, Saxe R | Human Brain Mapping |
| **95** | **2013** | Disorder-specific predictive classification of adolescents with attention deficit hyperactivity disorder (ADHD) relative to autism using structural magnetic resonance imaging. | Lim L, Marquand A, Cubillo AA, Smith AB, Chantiluke K, Simmons A, Mehta M, Rubia K | PloS One |
| **96** | **2014** | Abnormal autonomic and associated brain activities during rest in autism spectrum disorder. | Eilam-Stock T, Xu P, Cao M, Gu X, Van Dam NT, Anagnostou E, Kolevzon A, Soorya L, Park Y, Siller M, He Y, Hof PR, Fan J | Brain: A Journal of Neurology |
| **97** | **2014** | Structural alterations of the social brain: a comparison between schizophrenia and autism. | Radeloff D, Ciaramidaro A, Siniatchkin M, Hainz D, Schlitt S, Weber B, Poustka F, Bolte S, Walter H, Freitag CM | PloS One |
| **98** | **2014** | Oxytocin improves behavioural and neural deficits in inferring others' social emotions in autism. | Aoki Y, Yahata N, Watanabe T, Takano Y, Kawakubo Y, Kuwabara H, Iwashiro N, Natsubori T, Inoue H, Suga M, Takao H, Sasaki H, Gonoi W, Kunimatsu A, Kasai K, Yamasue H | Brain: A Journal of Neurology |
| **99** | **2014** | Altered network topologies and hub organization in adults with autism: a resting-state FMRI study. | Itahashi T, Yamada T, Watanabe H, Nakamura M, Jimbo D, Shioda S, Toriizuka K, Kato N, Hashimoto R | PloS One |
| **100** | **2014** | Lost for emotion words: What motor and limbic brain activity reveals about autism and semantic theory. | Moseley RL, Shtyrov Y, Mohr B, Lombardo MV, Baron-Cohen S, Pulvermuller F | NeuroImage |
| **101** | **2014** | Atypical perception of affective prosody in Autism Spectrum Disorder. | Gebauer L, Skewes J, Horlyck L, Vuust P | NeuroImage: Clinical |
| **102** | **2014** | Equivalent neural responses in children and adolescents with and without autism during judgments of affect. | Vander Wyk BC, Hoffman F, Pelphrey KA | Developmental Cognitive Neuroscience |
| **103** | **2014** | Oxytocin and vasopressin effects on the neural response to social cooperation are modulated by sex in humans. | Feng C, Hackett PD, DeMarco AC, Chen X, Stair S, Haroon E, Ditzen B, Pagnoni G, Rilling JK | Brain Imaging and Behavior |
| **104** | **2014** | Attenuation of the contingency detection effect in the extrastriate body area in autism spectrum disorder. | Okamoto Y, Kitada R, Tanabe HC, Hayashi MJ, Kochiyama T, Munesue T, Ishitobi M, Saito DN, Yanaka HT, Omori M, Wada Y, Okazawa H, Sasaki AT, Morita T, Itakura S, Kosaka H, Sadato N | Neuroscience Research |
| **105** | **2014** | Social perception in autism spectrum disorders: impaired category selectivity for dynamic but not static images in ventral temporal cortex. | Weisberg J, Milleville SC, Kenworthy L, Wallace GL, Gotts SJ, Beauchamp MS, Martin A | Cerebral Cortex (New York, NY, 1991) |
| **106** | **2014** | A comparison of neural correlates underlying social cognition in Klinefelter syndrome and autism. | Brandenburg-Goddard MN, van Rijn S, Rombouts SA, Veer IM, Swaab H | Social Cognitive and Affective Neuroscience |
| **107** | **2014** | Intact brain processing of musical emotions in autism spectrum disorder, but more cognitive load and arousal in happy vs. sad music. | Gebauer L, Skewes J, Westphael G, Heaton P, Vuust P | Frontiers in Neuroscience |
| **108** | **2014** | Direct Gaze Elicits Atypical Activation of the Theory-of-Mind Network in Autism Spectrum Conditions. | von dem Hagen EA, Stoyanova RS, Rowe JB, Baron-Cohen S, Calder AJ | Cerebral Cortex (New York, NY, 1991) |
| **109** | **2014** | Neural systems for cognitive reappraisal in children and adolescents with autism spectrum disorder. | Pitskel NB, Bolling DZ, Kaiser MD, Pelphrey KA, Crowley MJ | Developmental Cognitive Neuroscience |
| **110** | **2014** | Differential role of temporoparietal junction and medial prefrontal cortex in causal inference in autism: An independent component analysis. | Murdaugh DL, Nadendla KD, Kana RK | Neuroscience Letters |
| **111** | **2014** | Attribution of emotions to body postures: An independent component analysis study of functional connectivity in autism. | Libero LE, Stevens CE Jr, Kana RK | Human Brain Mapping |
| **112** | **2014** | Neural responses to emotional expression information in high- and low-spatial frequency in autism: evidence for a cortical dysfunction. | Corradi-Dell'acqua C, Schwartz S, Meaux E, Hubert B, Vuilleumier P, Deruelle C | Frontiers in Human Neuroscience |
| **113** | **2014** | Functional brain networks and white matter underlying theory-of-mind in autism. | Kana RK, Libero LE, Hu CP, Deshpande HD, Colburn JS | Social Cognitive and Affective Neuroscience |
| **114** | **2014** | Neural and cortisol responses during play with human and computer partners in children with autism. | Edmiston EK, Merkle K, Corbett BA | Social Cognitive and Affective Neuroscience |
| **115** | **2014** | Brain activity of adolescents with high functioning autism in response to emotional words and facial emoticons. | Han DH, Yoo HJ, Kim BN, McMahon W, Renshaw PF | PloS One |
| **116** | **2014** | Differentiating neural reward responsiveness in autism versus ADHD. | Kohls G, Thonessen H, Bartley GK, Grossheinrich N, Fink GR, Herpertz-Dahlmann B, Konrad K | Developmental Cognitive Neuroscience |
| **117** | **2014** | The control of automatic imitation based on bottom-up and top-down cues to animacy: insights from brain and behavior. | Klapper A, Ramsey R, Wigboldus D, Cross ES | Journal of Cognitive Neuroscience |
| **118** | **2014** | Inferring a dual-stream model of mentalizing from associative white matter fibres disconnection. | Herbet G, Lafargue G, Bonnetblanc F, Moritz-Gasser S, Menjot de Champfleur N, Duffau H | Brain: A Journal of Neurology |
| **119** | **2014** | Neural substrates of numerosity estimation in autism. | Meaux E, Taylor MJ, Pang EW, Vara AS, Batty M | Human Brain Mapping |
| **120** | **2014** | Neocerebellar contributions to social perception in adolescents with autism spectrum disorder. | Jack A, Morris JP | Developmental Cognitive Neuroscience |
| **121** | **2014** | Brain organization underlying superior mathematical abilities in children with autism. | Iuculano T, Rosenberg-Lee M, Supekar K, Lynch CJ, Khouzam A, Phillips J, Uddin LQ, Menon V | Biological Psychiatry |
| **122** | **2014** | Common and distinct neural features of social and non-social reward processing in autism and social anxiety disorder. | Richey JA, Rittenberg A, Hughes L, Damiano CR, Sabatino A, Miller S, Hanna E, Bodfish JW, Dichter GS | Social Cognitive and Affective Neuroscience |
| **123** | **2015** | Sex Differences in the Default Mode Network with Regard to Autism Spectrum Traits: A Resting State fMRI Study. | Jung M, Mody M, Saito DN, Tomoda A, Okazawa H, Wada Y, Kosaka H | PloS One |
| **124** | **2015** | Typical and atypical neurodevelopment for face specialization: an FMRI study. | Joseph JE, Zhu X, Gundran A, Davies F, Clark JD, Ruble L, Glaser P, Bhatt RS | Journal of Autism and Developmental Disorders |
| **125** | **2015** | Neural Mechanisms of Emotion Regulation in Autism Spectrum Disorder. | Richey JA, Damiano CR, Sabatino A, Rittenberg A, Petty C, Bizzell J, Voyvodic J, Heller AS, Coffman MC, Smoski M, Davidson RJ, Dichter GS | Journal of Autism and Developmental Disorders |
| **126** | **2015** | A greater involvement of posterior brain areas in interhemispheric transfer in autism: fMRI, DWI and behavioral evidences. | Barbeau EB, Lewis JD, Doyon J, Benali H, Zeffiro TA, Mottron L | NeuroImage: Clinical |
| **127** | **2015** | Synchrony between sensory and cognitive networks is associated with subclinical variation in autistic traits. | Young JS, Smith DV, Coutlee CG, Huettel SA | Frontiers in Human Neuroscience |
| **128** | **2015** | Abnormal Neural Activation to Faces in the Parents of Children with Autism. | Yucel GH, Belger A, Bizzell J, Parlier M, Adolphs R, Piven J | Cerebral Cortex (New York, NY, 1991) |
| **129** | **2015** | Trait-level temporal lobe hypoactivation to social exclusion in unaffected siblings of children and adolescents with autism spectrum disorders. | Bolling DZ, Pelphrey KA, Vander Wyk BC | Developmental Cognitive Neuroscience |
| **130** | **2015** | Sex differences in the neural basis of false-belief and pragmatic language comprehension. | Frank CK, Baron-Cohen S, Ganzel BL | NeuroImage |
| **131** | **2015** | Heterogeneity of neural mechanisms of response to pivotal response treatment. | Ventola P, Yang DY, Friedman HE, Oosting D, Wolf J, Sukhodolsky DG, Pelphrey KA | Brain Imaging and Behavior |
| **132** | **2015** | Gender-specific modulation of neural mechanisms underlying social reward processing by Autism Quotient. | Barman A, Richter S, Soch J, Deibele A, Richter A, Assmann A, Wustenberg T, Walter H, Seidenbecher CI, Schott BH | Social Cognitive and Affective Neuroscience |
| **133** | **2015** | Linked alterations in gray and white matter morphology in adults with high-functioning autism spectrum disorder: A multimodal brain imaging study. | Itahashi T, Yamada T, Nakamura M, Watanabe H, Yamagata B, Jimbo D, Shioda S, Kuroda M, Toriizuka K, Kato N, Hashimoto R | NeuroImage: Clinical |
| **134** | **2015** | Brain Mechanisms for Processing Affective (and Nonaffective) Touch Are Atypical in Autism. | Kaiser MD, Yang DY, Voos AC, Bennett RH, Gordon I, Pretzsch C, Beam D, Keifer C, Eilbott J, McGlone F, Pelphrey KA | Cerebral Cortex (New York, NY, 1991) |
| **135** | **2015** | Functional Organization of the Action Observation Network in Autism: A Graph Theory Approach. | Alaerts K, Geerlings F, Herremans L, Swinnen SP, Verhoeven J, Sunaert S, Wenderoth N | PloS One |
| **136** | **2015** | Inverse fluoxetine effects on inhibitory brain activation in non-comorbid boys with ADHD and with ASD. | Chantiluke K, Barrett N, Giampietro V, Santosh P, Brammer M, Simmons A, Murphy DG, Rubia K | Psychopharmacology |
| **137** | **2015** | Autonomic and brain responses associated with empathy deficits in autism spectrum disorder. | Gu X, Eilam-Stock T, Zhou T, Anagnostou E, Kolevzon A, Soorya L, Hof PR, Friston KJ, Fan J | Human Brain Mapping |
| **138** | **2015** | Altered resting perfusion and functional connectivity of default mode network in youth with autism spectrum disorder. | Jann K, Hernandez LM, Beck-Pancer D, McCarron R, Smith RX, Dapretto M, Wang DJ | Brain and Behavior |
| **139** | **2015** | Default mode network segregation and social deficits in autism spectrum disorder: Evidence from non-medicated children. | Yerys BE, Gordon EM, Abrams DN, Satterthwaite TD, Weinblatt R, Jankowski KF, Strang J, Kenworthy L, Gaillard WD, Vaidya CJ | NeuroImage: Clinical |
| **140** | **2015** | Changes in intrinsic connectivity of the brain's reading network following intervention in children with autism. | Murdaugh DL, Maximo JO, Kana RK | Human Brain Mapping |
| **141** | **2016** | Neuroanatomical Alterations in High-Functioning Adults with Autism Spectrum Disorder. | Eilam-Stock T, Wu T, Spagna A, Egan LJ, Fan J | Frontiers in Neuroscience |
| **142** | **2016** | Disruption of structural covariance networks for language in autism is modulated by verbal ability. | Sharda M, Khundrakpam BS, Evans AC, Singh NC | Brain Structure & Function |
| **143** | **2016** | The effect of perceptual expectation on repetition suppression to faces is not modulated by variation in autistic traits. | Ewbank MP, von dem Hagen EA, Powell TE, Henson RN, Calder AJ | Cortex; a journal devoted to the study of the nervous system and behavior |
| **144** | **2016** | Resting-State Time-Varying Analysis Reveals Aberrant Variations of Functional Connectivity in Autism. | Yao Z, Hu B, Xie Y, Zheng F, Liu G, Chen X, Zheng W | Frontiers in Human Neuroscience |
| **145** | **2016** | Connectivity-based parcellation increases network detection sensitivity in resting state fMRI: An investigation into the cingulate cortex in autism. | Balsters JH, Mantini D, Apps MA, Eickhoff SB, Wenderoth N | NeuroImage: Clinical |
| **146** | **2016** | Abnormalities of Inter- and Intra-Hemispheric Functional Connectivity in Autism Spectrum Disorders: A Study Using the Autism Brain Imaging Data Exchange Database. | Lee JM, Kyeong S, Kim E, Cheon KA | Frontiers in Neuroscience |
| **147** | **2016** | Under-reactive but easily distracted: An fMRI investigation of attentional capture in autism spectrum disorder. | Keehn B, Nair A, Lincoln AJ, Townsend J, Muller RA | Developmental Cognitive Neuroscience |
| **148** | **2016** | Language comprehension and brain function in individuals with an optimal outcome from autism. | Eigsti IM, Stevens MC, Schultz RT, Barton M, Kelley E, Naigles L, Orinstein A, Troyb E, Fein DA | NeuroImage: Clinical |
| **149** | **2016** | Diminished neural adaptation during implicit learning in autism. | Schipul SE, Just MA | NeuroImage |
| **150** | **2016** | Differences in neural activity when processing emotional arousal and valence in autism spectrum disorders. | Tseng A, Wang Z, Huo Y, Goh S, Russell JA, Peterson BS | Human Brain Mapping |
| **151** | **2016** | Resting State Functional Connectivity MRI among Spectral MEG Current Sources in Children on the Autism Spectrum. | Datko M, Gougelet R, Huang MX, Pineda JA | Frontiers in Neuroscience |
| **152** | **2016** | Noise Reduction in Arterial Spin Labeling Based Functional Connectivity Using Nuisance Variables. | Jann K, Smith RX, Rios Piedra EA, Dapretto M, Wang DJ | Frontiers in Neuroscience |
| **153** | **2017** | Neural Basis of Visual Attentional Orienting in Childhood Autism Spectrum Disorders. | Murphy ER, Norr M, Strang JF, Kenworthy L, Gaillard WD, Vaidya CJ | Journal of Autism and Developmental Disorders |
| **154** | **2017** | Differential Fairness Decisions and Brain Responses After Expressed Emotions of Others in Boys with Autism Spectrum Disorders. | Klapwijk ET, Aghajani M, Lelieveld GJ, van Lang NDJ, Popma A, van der Wee NJA, Colins OF, Vermeiren RRJM | Journal of Autism and Developmental Disorders |
| **155** | **2017** | Latent and Abnormal Functional Connectivity Circuits in Autism Spectrum Disorder. | Chen S, Xing Y, Kang J | Frontiers in Neuroscience |
| **156** | **2017** | Decoding versus comprehension: Brain responses underlying reading comprehension in children with autism. | Bednarz HM, Maximo JO, Murdaugh DL, O'Kelley S, Kana RK | Brain and Language |
| **157** | **2017** | Reduced Gray Matter Volume in the Social Brain Network in Adults with Autism Spectrum Disorder. | Sato W, Kochiyama T, Uono S, Yoshimura S, Kubota Y, Sawada R, Sakihama M, Toichi M | Frontiers in Human Neuroscience |
| **158** | **2017** | Atypical activation of action-semantic network in adolescents with autism spectrum disorder. | Knaus TA, Burns C, Kamps J, Foundas AL | Brain and Cognition |
| **159** | **2017** | Social-cognitive brain function and connectivity during visual perspective-taking in autism and schizophrenia. | Eack SM, Wojtalik JA, Keshavan MS, Minshew NJ | Schizophrenia Research |
| **160** | **2017** | Neural Mechanisms Underlying Conscious and Unconscious Gaze-Triggered Attentional Orienting in Autism Spectrum Disorder. | Sato W, Kochiyama T, Uono S, Yoshimura S, Toichi M | Frontiers in Human Neuroscience |
| **161** | **2017** | Neural networks underlying language and social cognition during self-other processing in Autism spectrum disorders. | Kana RK, Sartin EB, Stevens C Jr, Deshpande HD, Klein C, Klinger MR, Klinger LG | Neuropsychologia |
| **162** | **2017** | White Matter Integrity and Treatment-Based Change in Speech Performance in Minimally Verbal Children with Autism Spectrum Disorder. | Chenausky K, Kernbach J, Norton A, Schlaug G | Frontiers in Human Neuroscience |
| **163** | **2017** | Changes in intrinsic local connectivity after reading intervention in children with autism. | Maximo JO, Murdaugh DL, O'Kelley S, Kana RK | Brain and Language |
| **164** | **2017** | Comparative Multimodal Meta-analysis of Structural and Functional Brain Abnormalities in Autism Spectrum Disorder and Obsessive-Compulsive Disorder. | Carlisi CO, Norman LJ, Lukito SS, Radua J, Mataix-Cols D, Rubia K | Biological Psychiatry |
| **165** | **2017** | Autism Spectrum Disorder Related Functional Connectivity Changes in the Language Network in Children, Adolescents and Adults. | Lee Y, Park BY, James O, Kim SG, Park H | Frontiers in Human Neuroscience |
| **166** | **2018** | Transdiagnostic deviant facial recognition for implicit negative emotion in autism and schizophrenia. | Ciaramidaro A, Bolte S, Schlitt S, Hainz D, Poustka F, Weber B, Freitag C, Walter H | European Neuropsychopharmacology: The Journal of the European College of Neuropsychopharmacology |
| **167** | **2018** | What Are You Doing With That Object? Comparing the Neural Responses of Action Understanding in Adolescents With and Without Autism. | Pokorny JJ, Hatt NV, Rogers SJ, Rivera SM | Journal of Autism and Developmental Disorders |
| **168** | **2018** | Indices of repetitive behaviour are correlated with patterns of intrinsic functional connectivity in youth with autism spectrum disorder. | Traynor JM, Doyle-Thomas KAR, Hanford LC, Foster NE, Tryfon A, Hyde KL, Anagnostou E, Evans AC, Zwaigenbaum L, Hall GBC | Brain Research |
| **169** | **2018** | Action simulation and mirroring in children with autism spectrum disorders. | Wadsworth HM, Maximo JO, Donnelly RJ, Kana RK | Behavioural Brain Research |
| **170** | **2018** | Cortical responses to dynamic emotional facial expressions generalize across stimuli, and are sensitive to task-relevance, in adults with and without Autism. | Kliemann D, Richardson H, Anzellotti S, Ayyash D, Haskins AJ, Gabrieli JDE, Saxe RR | Cortex |
